# Schooling Increases Risk Exposure for Fish Navigating Past Artificial Barriers

**DOI:** 10.1371/journal.pone.0108220

**Published:** 2014-09-30

**Authors:** Bertrand H. Lemasson, James W. Haefner, Mark D. Bowen

**Affiliations:** 1 Department of Biology and Ecology Center, Utah State University (USU), Logan, Utah, United States of America; 2 Fisheries and Wildlife Resources Group, United States Bureau of Reclamation, Denver, Colorado, United States of America; Swansea University, United Kingdom

## Abstract

Artificial barriers have become ubiquitous features in freshwater ecosystems and they can significantly impact a region's biodiversity. Assessing the risk faced by fish forced to navigate their way around artificial barriers is largely based on assays of individual swimming behavior. However, social interactions can significantly influence fish movement patterns and alter their risk exposure. Using an experimental flume, we assessed the effects of social interactions on the amount of time required for juvenile palmetto bass (*Morone chrysops* × *M. saxatilis*) to navigate downstream past an artificial barrier. Fish were released either individually or in groups into the flume using flow conditions that approached the limit of their expected swimming stamina. We compared fish swimming behaviors under solitary and schooling conditions and measured risk as the time individuals spent exposed to the barrier. Solitary fish generally turned with the current and moved quickly downstream past the barrier, while fish in groups swam against the current and displayed a 23-fold increase in exposure time. Solitary individuals also showed greater signs of skittish behavior than those released in groups, which was reflected by larger changes in their accelerations and turning profiles. While groups displayed fission-fusion dynamics, inter-individual positions were highly structured and remained steady over time. These spatial patterns align with theoretical positions necessary to reduce swimming exertion through either wake capturing or velocity sheltering, but diverge from any potential gains from channeling effects between adjacent neighbors. We conclude that isolated performance trials and projections based on individual behaviors can lead to erroneous predictions of risk exposure along engineered structures. Our results also suggest that risk perception and behavior may be more important than a fish's swimming stamina in artificially modified systems.

## Introduction

Nearly 65% of surveyed rivers and their aquatic habitats are threatened by human activity and climate change, with extinction rates among fresh water fish species rivaling those of past geological events [1]. A wide variety of artificial structures pepper the earth's river systems and are essential for diverting water to meet the needs of human societies, such as providing municipal drinking water, creating hydropower, and supporting agriculture [2], [3]. Unfortunately, a single facility alone can impede or divert millions of fish per year [4] and the cumulative impacts of multiple facilities across the landscape have lead to ecosystem fragmentation and isolation in many systems [5]. Human manufactured disturbances can also exacerbate an animal's risk exposure if its life history strategy results in maladaptive behavior in novel settings [6], which can result in both immediate and long-lasting impacts on fitness [7], [8]. A prominent risk minimizing strategy undertaken by most fish species is to form into social groups or schools of varying coherence [9], yet we know little of this behavior's impacts on individual risk as fish are forced to navigate past artificial barriers in their environment.

Human barriers, such as dams, water diversion facilities and pumping stations can expose fish to a variety of dangers, including physical harm from collisions, exhaustion from swimming exertion [10]–[12], increased stress levels [13], and elevated predation [2], [14]. Juvenile fish are particularly susceptible to the risks posed by combinations of these stressors and suffer the highest mortality rates when navigating through artificial bottlenecks [3]. Even if individuals escape harm, many migratory species are on a tight physiological schedule (e.g., the ‘smolt window’ in Salmonids). Migratory fish species respond to environmental cues, such as photoperiod, temperature, and flow, and undergo physiological changes to prepare for their osmotic transition between freshwater and saltwater regimes [15]. The optimal navigation strategy is therefore to pass artificial barriers quickly in order to minimize the risk of any immediate threats [10], which would also reduce any migration delays that can have negative consequences on future stock success [15], [16].

Engineering and operational efforts predominantly rely upon estimates of average individual swimming stamina and behavior to expedite the safe passage of a region's fish. The design objective is to enable a fish's capacity to avoid flows or structural designs that would otherwise either impede its progress or increase its mortality risk [17], [18]. Traditionally, most assays on barrier exposure or individual swimming stamina are conducted with large juveniles or adults that are tested in isolation [17], [19]–[21]. Recent efforts have questioned the transferability of these empirical estimates to field conditions, pointing out that adaptive behaviors are not simply a function of individual physiological and biomechanical performance metrics [2], [22]. Experiments with groups of fish challenged with bypassing a barrier have shown large differences in their navigational performance relative to estimates reported from prior trials on individuals [22]. However, we know little of how social interactions affect individual swimming patterns under such conditions. This information would be particularly useful in defining movement parameters in agent-based modeling efforts that couple individual behaviors and environmental conditions to assess how animals navigate past engineered structures in their environment [23], [24].

Most juvenile fish form into groups of varying social and physical organization [9]. While this strategy has primarily evolved as a means to reduce the risk of predation, it can also effectively mitigate travel costs. Social cues can improve migration success by serving to average out individual directional uncertainties along a gradient, or migration route, [25], [26] and can enhance directional decision-making [27]. Organized formations may also convey net energetic benefits, such as reducing the drag that individuals experience [9], [28]–[30]. Despite all of these potential benefits, environmental context can alter the adaptive value of an ingrained behavior. For instance, if environmental conditions generate severe disorientation then solitary navigation is hypothesized to become preferable to a social navigation strategy, such as schooling in fish [31]. Similarly, deviations from optimal positions in schooling fish can be energetically costly and definitive empirical evidence to support any hydrodynamic advantage to schooling remains elusive [29]. While context may reshape the costs and benefits of social movement strategies, like schooling, this strategy should none-the-less directly impact the basic movement parameters related to the speed and orientation of animals on the move.

In this study we tested the hypothesis that social interactions alter the swimming behaviors of fish when they are forced to navigate past an artificial barrier. Palmetto bass, a *Morone chrysops* × *M. saxatilis* hybrid, were selected for their availability and propensity to display polarized schooling behavior when young. We began by determining individual swimming performance using ramped velocity tests to establish the water velocity necessary to challenge the average individual's swimming stamina (experiment I). Subjects were then released upstream of a behavioral barrier under solitary and social conditions to evaluate how neighbors altered individual swimming behaviors and, subsequently, impacted risk exposure (experiment II). We found that risk exposure varied dramatically between fish swimming by themselves or within schools, where risk was measured as the time taken to navigate successfully downstream past the barrier. A fine-scale kinematic analysis further revealed how individual behaviors varied under each treatment. We conclude by discussing how the observed patterns relate to existing theory and empirical evidence concerning fish swimming behavior.

## Materials and Methods

### Ethics Statement

All work was conducted within the U.S. Bureau of Reclamation's water lab in Denver, CO, and was included within BHL's dissertation work at USU. The BOR facility did not have an Institutional Animal Care and Use Committee (IACUC) in place and so all handling procedures followed the ethical standards outlined by the National Research Council [32], which also aligns with those of the American Fisheries Society [33].

### Fish husbandry

**Figure 1:**
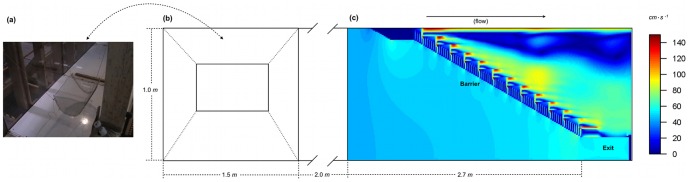
Top-down view of the experimental flume. The area included the swimming apparatus used in the stamina trials (a), the holding pen (b) and the louver-style hydraulic barrier (c), which leads to a 23 cm wide exit. Flume dimensions are in meters and flows within the flume were recreated using a Computational Fluid Dynamics model (CFD), with color profiles representing the speed of the water in 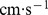 in section (c). The floating wire mesh cage (a) was suspended within area (b) with a series of cords. All stamina trials were done before the downstream barrier was installed. The upstream and down-stream boundaries of the holding area were enclosed with 1 × 1 cm^2^ wire screens.

Juvenile palmetto bass were obtained from Keo Fish Farms, AR, and maintained in flow-through cylindrical tanks (1.2 m in diameter, 1.4 m high), fed daily to satiation and experienced light: dark cycles typical of summer months in the northern hemisphere. Experiments were conducted in a 16 m flume (Figure 1). Water temperatures in the rearing pens and experimental flume varied between 19–20°C. A total of 283 fish were used in our experiments and all fish were used only once. Fish were drawn at random from the main population tank and transferred to the flume within an aerated bucket. After their trial each fish was allowed to rest in the aerated bucket until they resumed normal swimming behavior. Fish were then moved to a recuperation tank with the same dimensions and environmental conditions as that of the main population. If any fish was unresponsive for 24 h after their trial, due to either exhaustion or impingement against a retention screen, they were euthanized using MS-222 (Tricaine Methanesulfonate) at a prescribed dose of 400 mg/L.

### Experiment I. Individual swimming stamina

We determined the expected swimming stamina of our subjects using the critical swimming speed paradigm. Critical swimming speed, 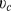, is measured by incrementally exposing individuals to increased water speeds (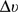) for fixed time intervals (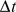) until the subject is exhausted [34].

The speed observed during the final successfully completed interval (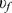) is then adjusted by the proportion of time spent in the subsequent interval in which exhaustion occurred (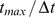):
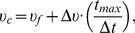
(1)


The performance trials estimate when an individual approaches its energetic limits, thereby providing a more conservative measure of its stamina. While fish are capable of swimming faster than this limit it generally requires them to transition to an anaerobically dominated phase and rapidly exhaust their oxygen supply [34], [35]. Individuals varied in body size so we standardized all 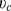 measurements to body length (i.e., fork length, FL) and exhaustion time (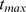) was recorded after a subject had collapsed against the rear screen for a second time. Fish ranged in size from 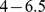 cm with a mean of 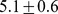 cm and weighed between 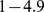 g with a mean wet weight of 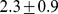 g (±SD). Pilot trials indicated that fish would occasionally exploit velocity refuges within the flume, so we adopted a novel approach and conducted our swimming trials in a wire cage that kept subjects suspended above the bottom and in the center of the flume (Figure 1a, b). The test cage was a half-cylinder (30 × 46 cm, 15 cm radius) composed of 0.6 cm wide wire mesh that was covered with a Plexiglas lid. The lid had a 10 cm baffle along its edges that caused the cage to rise with the water level during the velocity trials. An acoustic Doppler velocimeter was used to confirm that flow values within the cage did not differ from those in the main channel. A 10-minute adjustment period at 10 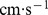 (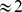 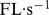) preceded each trial, after which the flow was incremented by 5 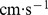 using 20 or 7 min intervals. Constrained time intervals were deemed necessary for filming during the subsequent barrier trials to preserve film, yet 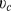 measurements based on short speed increments can inflate performance estimates [34]. We therefore compared both time intervals to determine how exposure time affected the expected swimming stamina of our subjects, 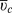. A total of 38 fish were used with 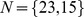 for the 20 and 7 min treatments, respectively, and performance data were recorded by an observer (see File S1, section SI-1 for further details).

### Experiment II. Solitary *vs*. schooling behavior

To determine if social interactions affect the time fish spend exposed to an artificial barrier we released fish upstream of a barrier either alone or in groups and filmed their swimming behavior from above. A louver was installed in the flume and angled to the oncoming water to guide fish to a bypass exit (Figure 1c). The barrier consisted of a series of vertical slats, each 2.5 cm apart and set perpendicular to the flow. A louver is designed to generate turbulence patterns meant to elicit avoidance maneuvers in fish, thereby passively guiding them towards an exit route. We focused on this type of behavioral barrier as part of an extension of earlier studies aimed at exploring how modeling fish behavior can inform management decisions at fish passage facilities (for further details on barrier history, design, and application see [19], [23], [36], [37]). Fish exposure time (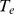) was measured as the time subjects spent within the barrier area, which equates to their risk of entrainment or impact in such artificial systems [10], [13]. We filmed our subjects using three Panasonic PV DV51 digital cameras that were installed above the barrier area so as to have overlapping fields of view. Each camera recorded at 29.97 frames per second and we stored our film segments on mini digital video tapes (60 min storage capacity). A Plexiglas sheet with a 15 cm baffle covered the test area to reduce any distortion from the water's surface.

Subjects were initially placed in a holding pen upstream of the diversion barrier either alone or in groups of 14. Fish ranged in lengths from 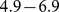 cm (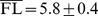 cm) and weighed between 1.4–5.0 g with a mean of 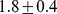 g (wet weight, ± SD). The number of subjects used in the social treatment conforms to the number needed to significantly influence movement decisions [27]. A 10-minute acclimation period followed with an initial flow of 10 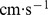, which seemed to be sufficient time for fish to cease any erratic swimming movements and display either station holding or minor movements. After this settlement period flows were then increased in 5 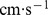 increments at 3–5 min intervals, which allowed the system to increase steadily until flows reached a maximum downstream speed of 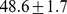 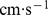 (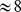 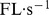), with an average cross-channel speed of 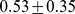 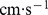 (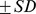). Maximum water velocity was selected based on our findings from the stamina trials and falls within the lower limit of flows recorded at a full-scale diversion facility that employs the same diversion design [37]. Flow increases here reflect a trade-off. Raising the water velocity too slowly risked premature exhaustion in our subjects from prolonged exposures, while raising the velocity too quickly risked creating a very turbulent system and disorientating the fish. Once the final water velocity had stabilized, the downstream screen of the holding pen was raised and individuals were allowed to drift passively towards the barrier area. We analyzed 14 of 21 solitary trials and 11 of 16 social trials. While a fixed number of fish were released in each social treatment (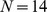), the fish would self-assemble into groups that varied in size over time. The size of a given group or school at any point in time was empirically defined from the observed dynamics (see File S1, section SI-2 and Analysis below for details). The unbalanced number of replicates stems from the removal of trials in which the subjects either displayed errant behaviors or remained upstream of the barrier beyond our designated recording limit (15 min, See File S1, section SI-3.1). Trials began when subjects entered the barrier area and ended when they either reached the exit or passed through the barrier's slats.

### Analysis

Our primary response variables were the critical swimming speeds (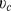) in experiment I and exposure times (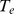) in experiment II. These data were skewed and therefore compared using the non-parametric Wilcox Rank Sum test. Secondary variables of interest described the swimming behavior of our subjects in experiment II and were extracted from our digital video recordings. Fish head and tail positions were manually tracked at 10 frames per second and each fish's centroid, 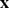, was estimated from these points to create a path from each field of view. Fish paths were then smoothed using a 5-point running median to improve velocity and acceleration estimates. Although individuals occasionally drifted vertically, movements were largely confined to the bottom of the channel (a behavior found in fish with similar morphologies and swimming gaits under comparable experimental conditions [12], [37]). Measurements within each field of view were converted from pixels to cm using a conversion metric for each axis (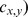 whose values were calculated from a virtual grid laid over each field of view during the post-processing stage (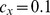 cm  pixel^−1^; 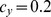 cm pixel^−1^).

The secondary variables used to characterize individual swimming behavior included: rheotaxis (orientation with respect to flow; downstream 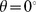), swim speed (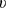), acceleration (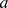), and turning angle, 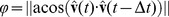. Any given turn is simply the difference between an individual's current heading and its past one, or 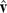 at 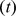 and 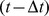, respectively. These turn angles fall between 0° and 180° as fish turn left or right in any given time step, so we report them as an absolute deviations in headings. Swim speeds were calculated by differencing fish centroids over time, correcting for water velocity and standardizing to average body length. Accelerations were then calculated by differencing the adjusted velocities. We controlled for spatial correlations among individuals in the social condition by randomly selecting a proxy fish from each replicate to represent socially influenced movement. Within each trial, fish were considered to be members of the same group when they were within 5 body lengths of one another; an interaction threshold empirically determined to balance the growth and decay rates in group membership observed in the data (Figure S1 in File S1). Fish released in groups often moved in and out of camera range, which prevented us from tracking the fate of any given individual for the entire duration of a trial. To avoid misidentifications and ensure path continuity, we randomly selected a representative sequence from each replicate for analysis, ensuring that a group of fish had distinct entry and exit points in the video sequence. In addition, groups in the social treatment were highly dynamic, continuously fragmenting and coalescing as they moved in and out of the filming area. We therefore investigated how these fission-fusion dynamics impacted overall risk exposure. Group cohesion or fusion was characterized by the mean observed size of our randomly selected groups within each trial 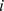 (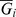) and we used the variability in these group sizes over time (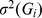) as a metric of the group's instability, or tendency to fragment, during a trial. These group-level metrics were recorded within each trial and related to the overall pattern in exposure times across trials. At the individual-level we investigated how nearest neighbor positions varied over space and time within groups. Nearest neighbor positions were based on the distance between a proxy fish 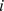 and its 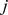 neighbors over time. Distance was calculated as the magnitude of the directional vector extending between position vectors, from fish 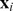 to fish 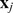, where 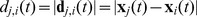. The bearing to a proxy fish's neighbor, 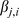, was the angular difference between the proxy's current heading, 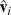, and its neighbor's bearing 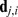, given as 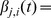 acos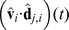. Time-series analyses were used to assess the reliability of all global averages reported in order to avoid spurious estimates from unsteady or biased trends in the data, as well as accounting for varying track lengths across trials (see SI-3). All analyses were conducted in R version 3.0.2. Laboratory experiments were originally conducted in late summer of 2003 and all analyses were repeated during the summer of 2013. Data supporting the table and figures are stored in the Knowledge Network for Biocomplexity repository, data package knb.480.1 (https://knb.ecoinformatics.org/).

## Results

### Individual swimming stamina

Standardized critical swimming speeds were statistically equivalent between the 20 min and 7 min intervals, being 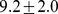 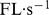 and 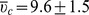 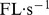, respectively (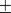 SD; Wilcoxon rank sum test, 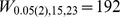, 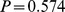; global mean  =  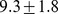 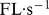). We recorded a dramatic decrease in post exercise mortality from 43% to 0% when trial increments declined from 20 to 7 min intervals, suggesting that prolonged exposure to elevated water velocities substantially decreased each fish's ability to recover from their trials.

### Solitary *vs*. schooling behavior

**Figure 2:**
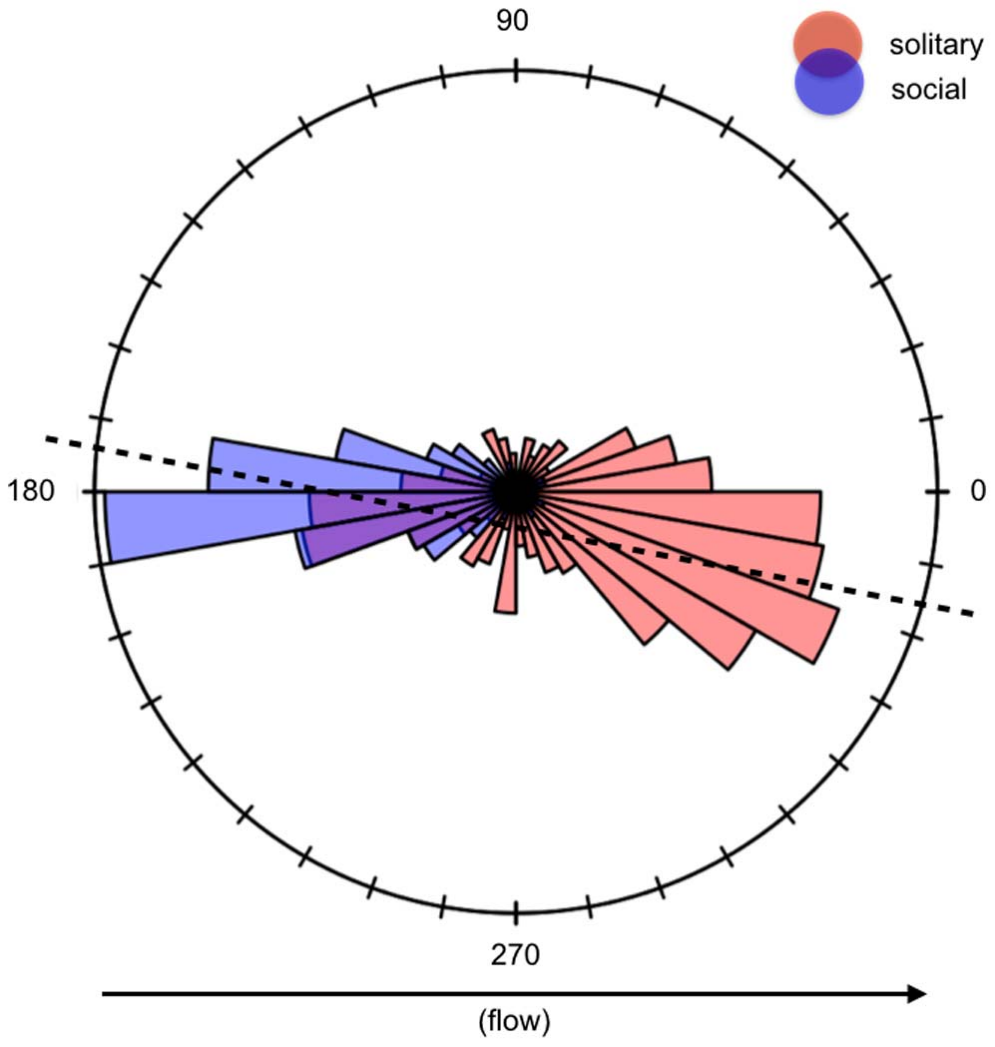
Circular histograms for the orientation patterns recorded under both solitary (red) and schooling (purple) conditions. Color modes are semi-transparent to show regions of overlap in the data from each condition. Schooling data represent only the proxy member's orientations from each replicate. The figure shows 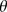 values pooled across all subjects within each category while in our statistical analyses we used the mean orientations from each replicate for both conditions. Upstream and downstream orientations are 180° and 0°, respectively. The dashed line represents the relative angle of the barrier with respect to the flow of water.

Solitary individuals tended to turn downstream and swim with the current (negative rheotaxis), while grouped fish predominantly faced upstream (positive rheotaxis; Watson's test, 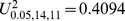, 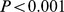, Figure 2, Table 1). These differences in preferred orientation occurred early on and remained steady over time (Figure S2 in File S1). Differences in turning angles between treatments were marginal, but significantly different from one another (Watson's test, 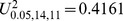, 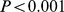; Table 1). Solitary individuals also swam at significantly slower swimming speeds than their schooling counterparts (Wilcoxon rank sum 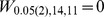; 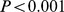), yet displayed stronger shifts in acceleration (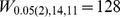, 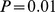, Table 1). The swim speeds of these solitary fish were unsteady and increased over time, as opposed to their accelerations, which remained steady as they moved downstream. In contrast, the proxy fish swimming in groups displayed steady speed and acceleration profiles throughout their tests (Figure S3 in File S1). Taken together these behavioral discrepancies led to a 23-fold increase in exposure times when fish were released in groups rather than alone (Table 1, Wilcoxon rank sum, 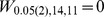, 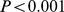). While exposure times under the social treatment were skewed and contained an outlier, omitting this trial had little impact on the median exposure times or any of the remaining kinematic data reported in Table 1. Interestingly, fish in either treatment were equally capable of safely reaching the exit (94% solitary; 98% social) and both treatment groups showed similar post exposure mortalities from either impacting the barrier or from impingement against a downstream retention screen (10% vs. 12%). In summary, a comparison of Table 1 and Figure 2 demonstrates that solitary fish turned with the current and so left the system quickly without having to exert themselves (low swim speeds). In contrast, fish traveling in groups faced into the current and effectively worked harder to hold their station (greater swim speeds), thereby substantially increasing their exposure times.

**Table 1:** Swimming characteristics of fish traveling either alone or within schools.

Metric	Solitary	Schooling	Units
Orientation, 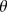	317.0 ± 48.9	187.3 ± 17.0	degrees
Turn angles, 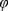	2.2 ± 6.9	0.5 ±1.4	degrees
Speed, 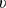	4.2 ± 1.2	7.6 ±0.4	FL s^−1^
Acceleration, 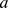	13.2 ± 5.2	6.8 ± 2.2	FL s^−2^
Exposure time, 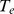	3.5 ± 0.6	82.0 ± 40.0	s

Data from schools are pooled from each replicate schools' proxy fish and do not represent group averages. We report the mean ± 1 SD for each circular metric (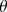 and 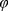). The linear metrics showed varying degrees of skewness, so we provide median ± median absolute deviation (MAD) for 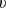, 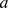, and 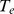. All metrics were significantly different between treatments.

### Schooling dynamics

**Figure 3:**
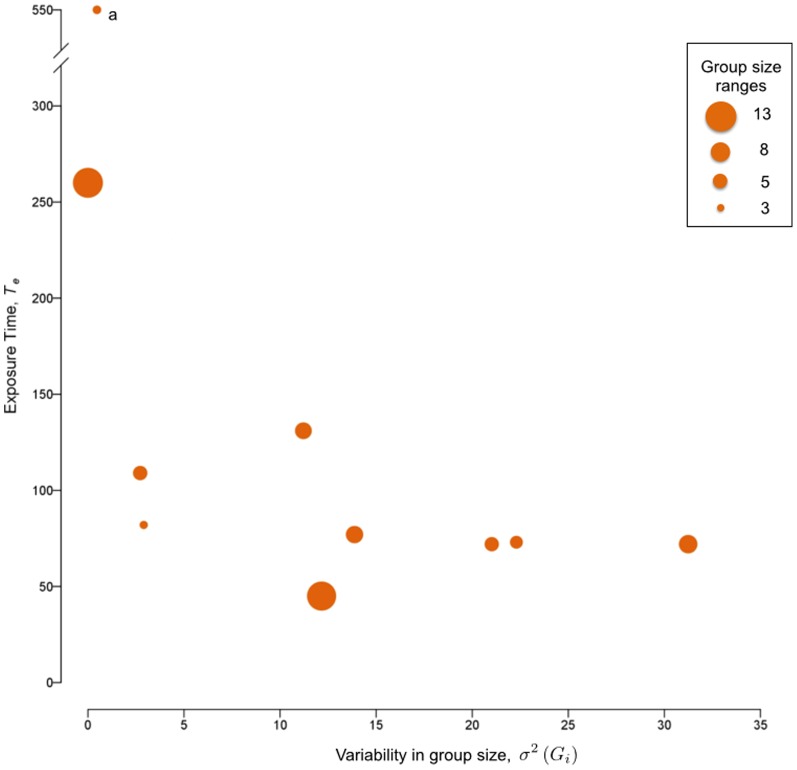
Non-linear correlation in exposure time with increasing variability in group size. Points represent the degree of variance observed across group sizes within each trial, while their diameters are scaled by the mean group size from those trials. While fish in the social treatment were released in groups of 14 individuals, these groups often fragmented and randomly selected representative groups varied in size both within and across trials. Overall, fish in the social treatment formed groups that ranged in size from 2–14 individuals with mean group sizes across trials ranging from 3.3 to 13. Solitary stragglers were also not uncommon (see SI-2). While extreme event (a) may represent a biologically plausible scenario, its absence does not detract from the relationship between variance in group size and exposure time.

**Figure 4:**
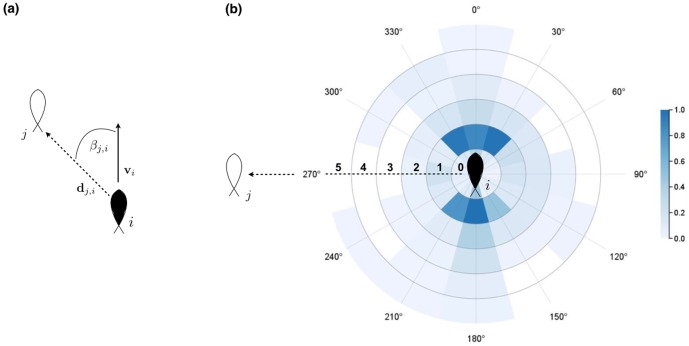
Nearest neighbor distributions within schools based on data pooled across all observations (replicates and time). Neighbor positions are taken from the perspective of each group's proxy fish. The distance, 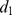, and bearing, 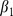, between a subject and its nearest neighbor (a) are presented in circular sections of 1 body length increments (FL) and binned by 30° intervals (b). Color values represent the normalized frequency of observations per bin and range from 0 (white) to 1 (dark blue). The highest concentration of nearest neighbors fall within 1 FL of the proxy fish, which tended to trail their leading neighbors at angles ranging from ±30° from their heading.

Fish released in groups displayed varying degrees of cohesion within and across trials, splitting and remerging as they moved in and out of camera range. Exposure time was not significantly correlated with mean group size (Spearman rank-correlation; 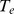 × 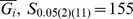; 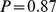), but was strongly correlated with group instability (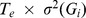, 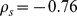, 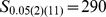; 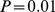). Figure 3 shows how overall exposure times were negatively correlated with increasing group instability, while no distinctive pattern arose with respect to the average size of the groups (where mean group size is shown by the diameter of the points). Retaining the outlier in exposure times (Figure 3) changes neither the direction nor the significance of either of these group-level associations. At the individual-level we found no correlations between either the nearest neighbor distances or bearings with regards to overall exposure time. Nearly all of the proxy fish chosen from each group (10/11) maintained a steady and close association with their nearest neighbors (median 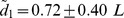; ± MAD) and predominantly trailed these neighbors at bearings between 30° and 330° (Figure 4). We found no evidence of any temporal trends in nearest neighbor positions or bearings, so while average trends in 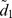 and 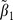 fluctuated over time these processes remained relatively steady (see Figures S4 and S5 in File S1). There was, however, greater variability in 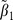 values over time, which is not unexpected. As fish speed up or slow down even minor shifts in the relative distances between individuals can result in larger changes in bearings, as a fish's closest neighbor can suddenly shift orthogonally, or from front to back.

## Discussion

Fish released in groups up-stream of a diversion barrier formed schools and took longer to navigate downstream past the barrier than fish that were released alone. Schooling has evolved as an adaptive behavior largely because it decreases the immediate risks that fish face, whether it be by diluting the threat of predation, or reducing directional uncertainty [9]. Anthropogenic structures in rivers and streams are dangerous places for juvenile fish [2], [11], [12], [14], [15], [17] and their risk of mortality increases with their exposure to these environments [10]. Additionally, the immediate threats that fish face when navigating past diversion barriers (e.g., collision, exhaustion, stress) can also be compounded by indirect costs, such as detracting from more profitable opportunities like searching for resources or mates in other areas (e.g., the risk allocation hypothesis) [38]. Our results demonstrate that the long held practice of managing water diversion facilities based on individual fish swimming stamina [17], [19]–[21] can significantly underestimate how long fish will linger along a diversion barrier.

Results from our stamina trials indicated that a current of 48.6 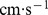 approached the upper limits of our subjects' swimming stamina (46 – 49 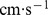). Under these circumstances our subjects would generally not be expected to hold their station for extended periods. Yet, water velocity alone is a poor predictor of fish residency times within and across species [23], [24], suggesting that individual behavior may either reduce or exacerbate exposure times. As fish move downstream with a current even minor trajectory deviations brought about by obstacle avoidance maneuvers can change the time they take to traverse an area. For instance, a biased-random walk model, parameterized to reflect fish swimming speeds, demonstrates that the expected exposure time of a particle can increase by a factor of 5 simply by accounting for self-propulsion and obstacle avoidance behavior [37]. Within the relatively uniform flows found in our flume a passive particle released from the holding pen would take approximately 12 s to drift downstream past the barrier if it traveled in a straight line towards the exit. Although the shorter exposure times observed with the solitary individuals may fall within some, albeit large, margin of error of this expectation, the 23 fold increase in exposure time by those fish released in groups represents a substantial deviation from a purely hydraulic prediction. Group members swam at twice the speed of their solitary counterparts, showed less erratic swimming behavior, and were predominantly moving upstream. To understand how such pronounced differences could have arisen requires a brief review of the physical, energetic, and behavioral factors that may influence the observed patterns and our ability to draw broader conclusions from them.

Palmetto bass share diet and habitat preferences with their maternal species in the wild and integrate themselves into striped bass spawning migrations [39], [40], which suggests at least parallels in their movement behaviors under natural conditions. Palmetto bass also display an innate ability to form polarized schools when young and so they provide a pragmatic means to study the physical implications of coordinated motion. Hatchery-bred hybrids are likely to suffer the same biases displayed by hatchery fish, such as ecological naïveté with respect to foraging or predator avoidance. Yet, despite such limitations hatchery reared fish have proven useful in studying the fundamental mechanics of collective motion, yielding insights into topics ranging from gradient detection [41] to social learning and decision-making [42], [43]. These emergent behaviors stem from the basic physical mechanics of coordinated motion, which can have ecologically relevant impacts in wild populations. For instance, the speed and distance travelled by Chum (*Oncorhynchus keta*) and Pink (*O. nerka*) salmon generally increases with the size of the schools they form [44]. As a group grows in size, or density, the frequency of local interactions increases and can result in directional feedbacks that reduce overall turning variability among individuals [25], [44]. Similar behavior has been documented in locusts [45], demonstrating that the underlying physical mechanics can have an impact that transcends taxa and context. Care should none-the-less be taken in interpreting our results beyond the physical ramification of displaying polarized schooling, such as the potential ecological reasons that influence when or why a fish species will school.

Data also suggests that the size of the group may play a role. For example, the ability of Atlantic salmon (*Salmo salar*) and American Shad (*Alosa sapidissima*) to navigate through bypass weirs decreases with increases in the size of the schools they form, with the majority of fish breaking off from larger groups and passing their respective barriers in pairs or alone [46]. Fish exposure time in our study was negatively correlated with group instability, suggesting that fish in groups may have been passing through the system more quickly as social interactions degraded. Taken together these associations highlight the need to test for a group size effect to determine if there is a causal relationship and, if so, determine how this factor can influence management strategies.

Fish traveling in groups in our study followed leading neighbors more closely than those swimming alongside them (Figure 4b). This pattern can arise from two alternative, yet not mutually exclusive, ecological mechanisms. In the early 1970's, Daniel Weihs theorized that hydrodynamic advantages can arise predominantly from three means: channeling effects between parallel neighbors, flow sheltering behind neighbors, or by capitalizing on the energy generated by trailing vortices [28], [29]. Channeling effects are enhanced when individuals are separated by 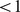 body length and these effects decay rapidly with distance [28], [29]. Our subjects tended to position themselves further than this optimum, yet predominantly followed or led neighbors at bearings that closely corresponded with those predicted by Weihs' theoretical diamond configuration (±30°). Moreover, these patterns were not only spatially structured, but also remained relatively steady over time. Taken together these patterns suggest that potential hydrodynamic gains were theoretically plausible and, if present, more likely to stem from either flow avoidance or vortex capturing than from channeling effects between parallel swimmers.

Recent evidence has shown that leader positions are significantly correlated with an individual's metabolic scope (MS), with spatial positioning from a school's front to rear being negatively correlated with the constituents' MS [47]. Individual fish have also been shown to display less muscle activity when drafting behind the vortices shed from a stationary object [29], so it is certainly possible for individuals to take advantage of similar physical mechanisms that may arise within a school. However, we continue to find opposing predictions from theoretical efforts exploring the hydrodynamic benefits of schooling [48], [49]. The leader-follower dynamics seen in our subjects are found outside of advection dominated systems [50]–[52] and may therefore arise for ecological reasons beyond any hydrodynamic advantage. Leadership positions are typically ephemeral in fish groups as they generally lack a social hierarchy [52] and are more commonly attributed to foraging information [50]–[52], aerobic capacity [47] and risk taking [9]. All of our individuals were fed to satiation, were naïve as to the location of the exit route, and either rarely or never exceeded their expected critical swimming speed. So, while the positional patterns in Figure 4b provide evidence to support some of Weihs' assumptions, another plausible explanation is that risk perception can play an equal, if not more important, role in such artificial environments.

Consider that ‘leaders’ within groups can either possess information, or merely display bold tendencies [50], [53]. Bold animals typically commit quickly to reactionary behaviors, such as fleeing or attacking, and show less variability in their movements than the typical individual [54]. We might therefore expect bold individuals to move quickly and decidedly downstream past the barrier, as displayed by the solitary travelers. However, our solitary subjects showed greater variability in their turning angles and accelerations than those fish released in groups. Such elevated variability is better associated with shy or risk-averse individuals than with bold ones [54], which suggests that solitary individuals were simply more skittish than those released in groups and moved through the system quickly due to their orientation in the water. While social interactions affect an individual's reaction to stressful scenarios (e.g., predators), such interactions also significantly impact stress responses at the behavioral and metabolic level [55], [56]. We can simplify this metabolic argument by considering that in the simplest models of collective behavior, individuals average the motion of their neighbors in order for any degree of coordinated or cohesive motion to emerge [9]. This elementary assumption alone invariably leads to a dampening of individual movement variability, which may, in turn, indirectly decrease metabolic rates caused by erratic movements. Regardless of whether fish traveling in groups were able to work less to remain upstream of the barrier, their interactions significantly reduced any propensity for erratic movements.

In conclusion our findings demonstrate that schooling can enhance individual risk exposure for fish swimming through artificial environments. Future efforts would benefit from exploring how group size, hydraulic gradients, and structural complexity influence schooling behavior and individual exposure times along manufactured structures and obstructions in aquatic systems. While a thorough understanding of the fluid dynamics within schools remains out of reach, including basic social interactions when modeling animal movements may improve our ability to provide reliable risk assessments.

## Supporting Information

File S1 **This file contains supporting information for the article and also contains Figure S1–Figure S5.** Figure S1, The influence of interaction range, r, on the size (a) and number (b) of groups observed. Figure S2, Time series of rheotactic patterns observed in the solitary and social travel conditions. Figure S3, Time series of observed swimming velocity and acceleration in both the solitary and social conditions. Figure S4, Time series of nearest neighbor values, *d*_1_. Figure S5.
(PDF)
