# Supplementary material for: Schooling Increases Risk Exposure for Fish Navigating Past Artificial Barriers
Source: PLoS One. 2014 Sep 30;9(9):e108220. doi: 10.1371/journal.pone.0108220 (PMC4182462; doi:10.1371/journal.pone.0108220)
Supplement: File S1 — This file contains supporting information for the article and also contains Figure S1–Figure S5. Figure S1, The influence of interaction range, r, on the size (a) and number (b) of groups observed. Figure S2, Time series of rheotactic patterns observed in the solitary and social travel conditions. Figure S3, Time series of observed swimming velocity and acceleration in both the solitary and social conditions. Figure S4, Time series of nearest neighbor values, d 1. Figure S5. (PDF) [file pone.0108220.s001.pdf]

Supplemental Information:

Schooling increases risk exposure for fish navigating past artificial barriers.

Bertrand H. Lemasson [1,2,*], James W. Haefner [1], Mark D. Bowen [3,4]
[1] Department of Biology and Ecology Center, Utah State University (USU), Logan, UT, 84321, USA
[2] Present address: Environmental Laboratory, U.S. Army Engineer Research & Development Center, Santa Barbara, CA, 93101, USA
[3] Fisheries and Wildlife Resources Group, US Bureau of Reclamation, P.O. Box 25007, 6th and Kipling, Building 67, Denver, CO, USA
[4] Present address: Turnpenny Horsfield Assoc., Ashurst Lodge, Ashurst, Southampton, Hampshire, SO40 7AA, UK

∗ Correspondence: brilraven@gmail.com

Contents

SI 1. Critical swimming trials **2**

SI 2. Interaction range **3**

SI 3. Temporal patterns **4**

SI 3.1 Rheotaxis, speed and acceleration 5

SI 3.3 Nearest neighbor positions 7

References **8**

SI 1. Critical swimming trials

Pilot trials demonstrated that fish would occasionally exploit low velocity zones along the edges of the channel, displaying relatively few tail beats to remain stationary for extended periods despite elevated water velocities. We controlled for this behavior by placing solitary fish within a test domain that kept them suspended within the center of the water column. The cross-sectional area of our subjects were less than 5% than that of the cage, so critical swimming estimates did not have to be adjusted for any solid blocking effects [1]. The wired cage also served to reduce wall effects that can bias performance estimates [2]. We found no records of critical swimming speeds for either palmetto bass or white bass in the literature. The minimum number of fish used to estimate v_c were therefore based on the range of values reported from studies using striped bass [3–5], with the number of replicates for the $\Delta t = 20$ min treatment being increased to offset mortalities. However, including data from those fish that later expired in these trials did not affect the results presented in the main text. Relative v_c values in striped bass generally range between 1.5 - 5 $\mathrm{FL} \cdot \mathrm{s}^{-1}$, with recorded values for larger striped bass falling between 3.3 $\mathrm{FL} \cdot \mathrm{s}^{-1}(\overline{\mathrm{FL}} = 24$ cm) to 4.8 $\mathrm{FL} \cdot \mathrm{s}^{-1}(\overline{\mathrm{FL}} = 9.7$ cm) [3, 6–8]. Our elevated v_c values could be explained by either hybrid vigor or the fact that swimming stamina changes non-linearly with body size as individuals grow. Relative swimming stamina ($\mathrm{FL} \cdot \mathrm{s}^{-1}$) tends to decrease with body size while absolute swimming stamina ($\mathrm{cm} \cdot \mathrm{s}^{-1}$) tends to increase [6]. High relative v_c values are therefore not unexpected in smaller fishes and a wide range of responses is found across species and size classes (6–18 $\mathrm{FL} \cdot \mathrm{s}^{-1}$) [3, 4, 6–9].

The duration (Δt) and magnitude (Δv) of our velocity increments were chosen to reduce the potential for restlessness after each velocity augmentation. Such restlessness stems from spikes in the subject's oxygen consumption rate due to stress that can cause premature fatigue and can bias stamina trials [8]. Signs of restless behavior quickly subsided in all subjects, indicating that the short acclimatization period was sufficient to reduce stress and minimize

any erroneous metabolic costs associated with spontaneous swimming behavior. Subjects would also occasionally display exploratory behavior when flows were increased, moving to either the front, back, or sides of the cage. However, most subjects would then settle on a position near the front of the cage just behind the screen. Such changes in swimming behavior during velocity increments are quite common and their prevalence is used to argue against short time intervals in determining swimming stamina [8]. Screen-drafting behavior was also observed in tangential experiments where individuals would often swim right behind any up-stream screen. Despite our precautions, the primary purpose of the critical swimming trials should generally be interpreted as a relative estimate of our subject's swimming stamina, rather than an accurate representation of their maximum energetic efficiency [8].

SI 2. Interaction range

Fish released in the social treatment displayed a wide range of behaviors, as individuals frequently joined and left groups. How to parsimoniously define what constitutes a school or 'group' under such circumstances is an open question. We approached the problem by using an equivalence algorithm in which group membership was defined by an interaction range, r [10,11]. We then fixed this free parameter according to the value where the rate of growth in group size begins to decay with increasing values of r. In our data this transition point is qualitatively observed around a range of 5 body lengths (Fig. S-1a). At this interaction range we also find that the median number of groups across trials has approached unity (Fig. S-1b), but from Fig. S-1a we know that this is not simply because group size has saturated (i.e., a case where the number of observable groups cannot grow anymore because r is large enough to encompass all fish). Fixing $r = 5$ FL therefore appears to equitably reflect the dynamic nature of our data, with group sizes displaying a bi-modal distribution in which individuals are almost as likely to become separated from their neighbors as they are to remain together (Fig. S-1c).

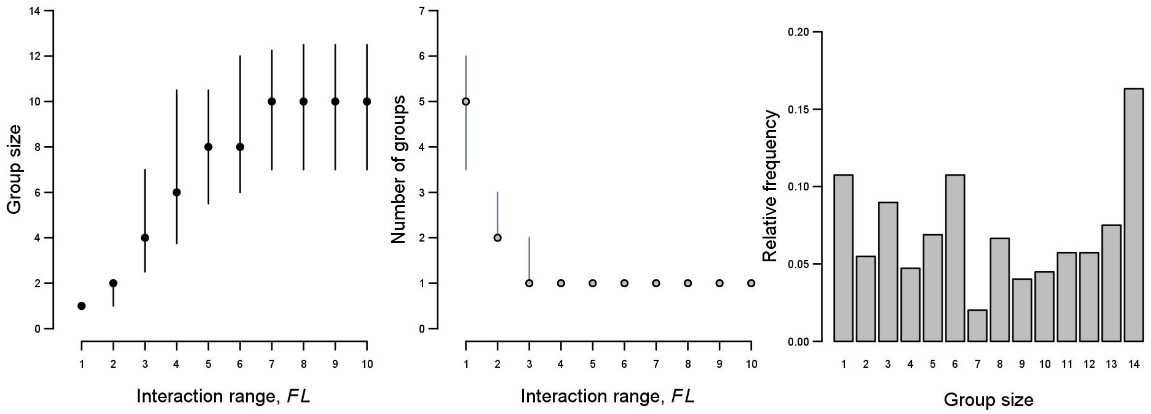

Figure S-1. The influence of interaction range, r, on the size (a) and number (b) of groups observed. Data in (a) and (b) represent median values ± 25% and 75% quantiles. At $r = 5$ FL fish released in groups display a bimodal distribution in group sizes (c). Interaction length is normalized to body length.

SI 3. Temporal patterns

To determine if the average swimming behaviors of our subjects remained steady over time we tested whether or not the metrics used to describe their movements could each be modeled as a stationary process. Specifically, a second-order stationary process where only the first two moments of the stochastic process need to remain constant. Most real world data, from biology to finance, fail strict definitions of stationarity, so for our purposes it is sufficient to follow the convention of adopting the weaker definition. We explored how the average fish's rheotaxis, speed, and acceleration each changed over time, as well as the distance and bearing to a fish's nearest neighbor within a group ($z : \{\theta,\ v,\ a,\ d_1,\ \beta_1\}$). For each metric z we characterized the average observed response as the median value taken across replicates at each point in time, $\tilde{z}_t$. We used the Kwiatkowski-Phillips-Schmidt-Shin (KPSS) method to test the null hypotheses H_o that a given variable $\tilde{z}_t$ is either I(0) stationary around an initial level (test statistic $\hat{\eta}_\mu$) and/or a discernible trend ($\hat{\eta}_\tau$). I(n) refers to the order of integration, or the number of times the series needs to be differenced to achieve stationarity. We then used the augmented Dickey-Fuller (DF) method to either substantiate or challenge the KPSS results. The DF test also served to determine if the data could be modeled as an I(1) process

(H_o) and be explained by a random walk with or without drift. Concluding that $\tilde{z}_t$ can be modeled as a stationary process would entail KPSS rejecting its null and DF failing to do so. Critical values for the DF models, including terms for the H_o (unit root [1], τ), trend (ϕ_T) or drift (ϕ_D) were taken from [12]. In the case where the results from the KPSS and DF tests conflicted, our interpretation relied upon the KPSS test since it has more power. All lag values k fell within $k = N \cdot (f/100)^{0.25}$, where f is the number of frames in the time series and N sets the minimum/maximum allowable lags ($N : \{4, 12\}$). Within these bounds lag selection was based on stopping at the last serially significant lag and verifying the selection based on a visual inspection of the partial auto-correlations seen in the residuals. Analyses were done using the **tseries** and **urca** package in R.

SI 3.1 Rheotaxis, speed and acceleration

Rheotactic patterns differed greatly between the solitary and social treatments and these tendencies persisted over time. Preferred orientations generally occurred before the subjects reached the barrier in both treatments, with few instances of behavioral switching in either group (Fig. S-2). Most solitary fish had already turned to swim with the current before entering the barrier section of the channel. Several solitary trials showed signs of errant behavior and were omitted. Errant behavior included signs of being startled by something, turning with the current and immediately bolting downstream out of the holding pen, or exploiting a velocity shelter and remaining in the holding pen. Only fish that showed no signs of these errant behaviors were retained for analysis. Several trials in the social treatment were also excluded because the subjects remained upstream of the barrier for longer than 15 min, which was our designated cutoff limited by our film stock. The average (median) trends in the temporal patterns in rheotaxis were sine transformed to circumvent issues of

[1] *Unit root*– refers to the root value of a stochastic process's characteristic equation. Consider a process z that varies over time and is characterized by the auto-regressive (ar) model, $z_t = c + \phi z_{t-1} + \epsilon_t$, where the trajectory of the process depends upon ϕ. If $|\phi| = 1$ the process has a unit root. If $|\phi| \leq 1$ shocks to the system induced by ϕ decay over time, whereas they grow or 'explode' when $|\phi| > 1$. A series with a unit root is considered to be a first order integrated process, I(1) [11].

circularity [13]. Average values of the transformed rheotactic data showed no indication of a trend and were level stationary under both solitary ($\hat{\eta}_\mu = 0.22, P = 0.1$; $\tau = -4.02, P = 0.01$) and social conditions ($\hat{\eta}_\mu = 0.07, P = 0.1$; $\tau = -3.59, P = 0.02$).

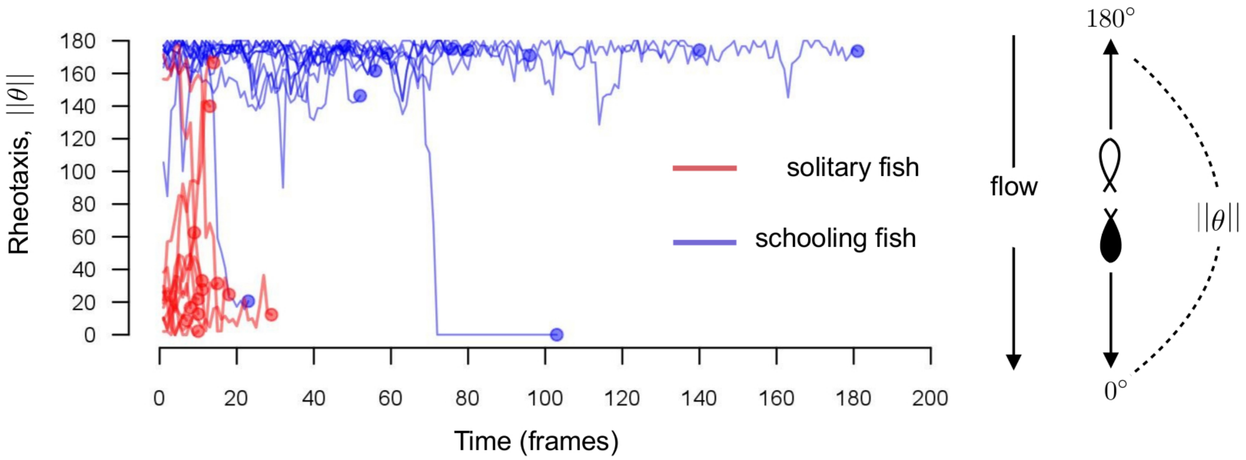

Figure S-2. Time series of rheotactic patterns observed in the solitary and social travel conditions. An orientation of 0° faces downstream. Final positions from each recorded path are marked by a circle.

Solitary fish speeds were unsteady, increased over time and failed tests of stationarity for both the level (KPSS: $\hat{\eta}_\mu = 0.65$, $P = 0.02$) and trend tests ($\hat{\eta}_\tau = 0.16$, $P = 0.03$; Fig. S-3). The DF tests supported this result ($\tau = -1.39$, $P = 0.82$), yet the trend and drift parameters were not significant ($\phi_T = 1.62$, $\phi_D = 1.70$), which suggests that the speeds of solitary fish were best modeled as a random walk. The KPSS and DF test results for the accelerations of these subjects contradicted one another, with KPSS indicating that the process was I(0) stationary ($\hat{\eta}_\mu = 0.33$, $P = 0.1$), while the DF test failed to reject its null hypothesis of a unit root ($\tau = 0.48$, $P = 0.99$). Differencing the speed data does not result in a stationary process, which one may expect given the relationship between velocity and acceleration. It is likely that the tendency of solitary fish to turn and swim with the current contributed to the decoupling of their speed and acceleration profiles. Those fish traveling under social conditions showed speed and acceleration profiles that were both I(0) stationary, a result that both tests agreed upon. In the $\tilde{v}_t$ time series from the social treatment $\hat{\eta}_\mu = 0.21$ ($P = 0.1$) and $\tau = -3.58$ ($P = 0.04$), while for the $\tilde{a}_t$ series for these fish $\hat{\eta}_\mu = 0.08$ ($P = 0.1$) and $\tau = -4.23$ ($P = 0.01$).

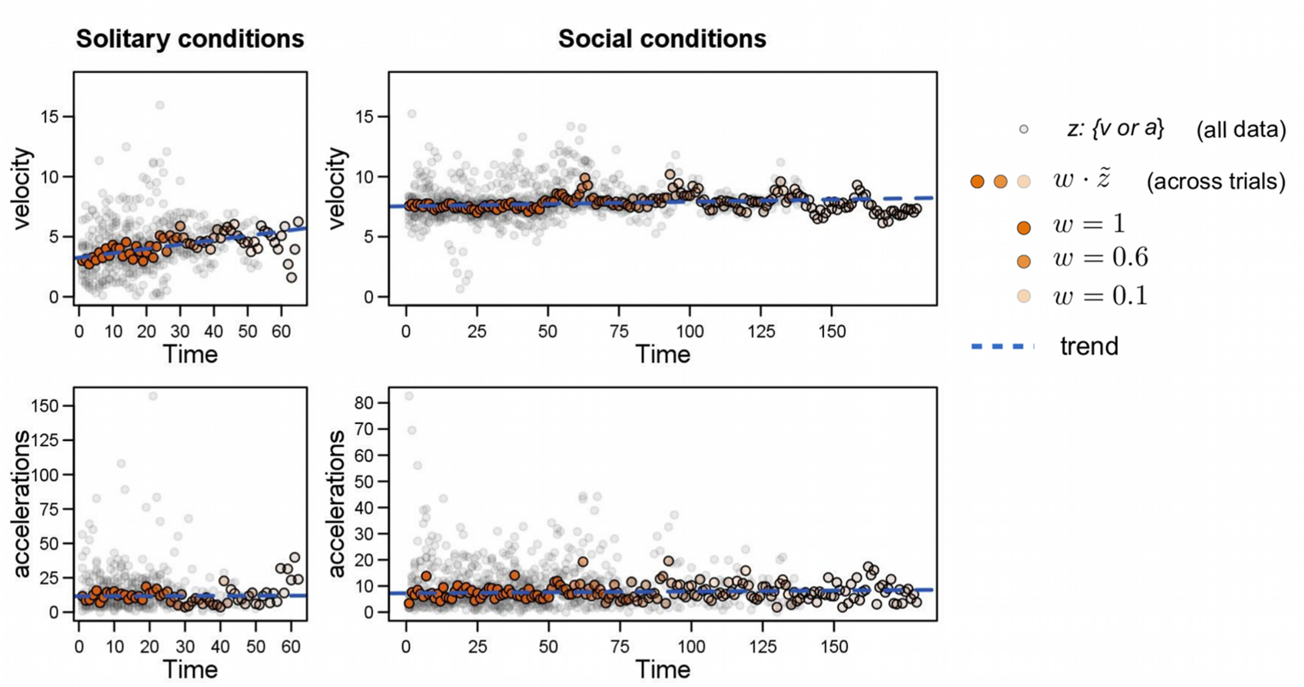

Figure S-3. Time series of observed swimming velocity and acceleration in both the solitary and social conditions. Median values for both metrics ($\tilde{z} : \{v, a\}$) are taken across replicates at each frame (orange points) from the raw data (grey). Median values are weighted by w to reflect the relative number of replicates used in their calculation. Data are uniformly weighted in the stationarity and unit root tests.

SI 3.3 Nearest neighbor positions

The distance and bearing to a fish's nearest neighbor for those fish traveling in groups were taken from the proxy fish's perspective and are the same data used to generate Fig. 4b in the main text. A single trial was omitted from this analysis because its proxy separated from its group relatively quickly ($\approx$ 40 time steps into the sequence; Fig. S-4). Models fit to the $\tilde{d}_1$ data showed no significant trend over time and were I(0) stationary around their initial level (KPSS, $\hat{\eta}_\mu = 0.38$, $P = 0.09$; DF, $\tau = -4.03$, $P = 0.01$). Nearest neighbor bearings (Fig. S-4a) were sine transformed and were not I(0) stationary around their initial level (KPSS, $\hat{\eta}_\mu = 0.62$, $P = 0.02$; DF, $\tau = -2.86$, $P = 0.23$), yet differencing y values once generated a stationary process (KPSS, $\hat{\eta}_\mu = 0.05$, $P = 0.1$; DF, $\tau = -3.91$, $P = 0.02$). While trends in bearing data appear to become more volatile over time this is primarily due to the limited number of replicates that lasted longer than 100 frames (2 replicates lasting 10 s).

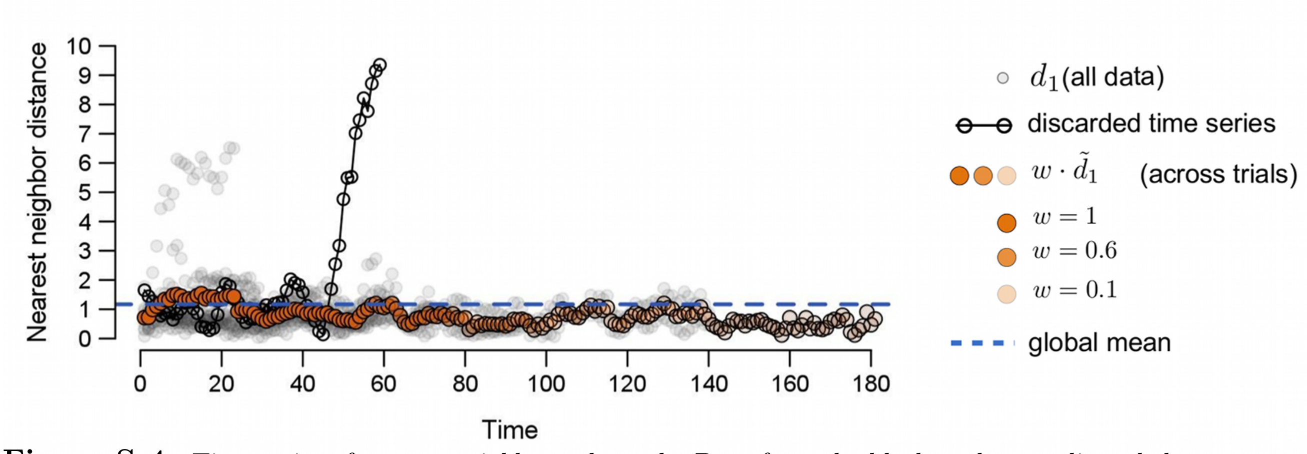

Figure S-4. Time series of nearest neighbor values, d_1. Data from the black path were discarded because the proxy fish became isolated from its group well before the average recorded path length. Data are visualized as in Figure S-3.

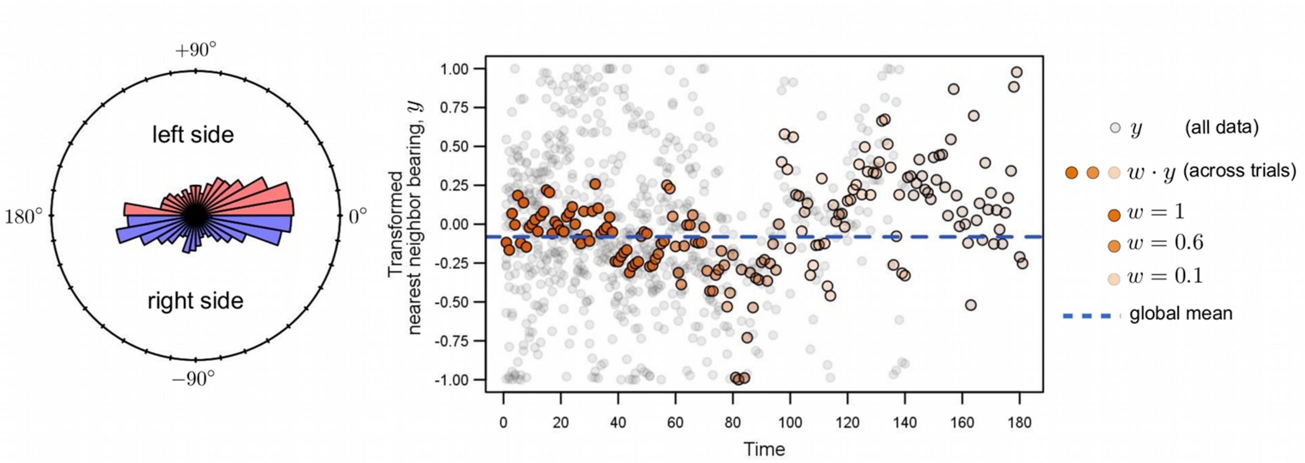

Figure S-5. Distribution of nearest neighbor bearings, β_1, pooled across all observations (a) and the time series of their linearized representation. Left and right bearings (red and purple; a) become positive and negative when transformed (b). Data are visualized as in Figures S-3 and S-4.

References

1. Brett J (1964) The respiratory metabolism and swimming performance of young sockeye salmon. J Fish Res Board Can **21**: 1183-1226.

2. Webb P (1993) The effect of solid and porous channel walls on steady swimming of steelhead trout *Oncorhynchus mykiss*. J Exp Biol 178: 97-108.

3. Freadman M (1979) Swimming energetics of striped bass (*Morone saxatilis*) and bluefish (*Pomatomus saltatrix*): gill ventilation and swimming metabolism. J Exp Biol 83: 217-230.

4. Hurst T, Conover D (2001) Activity-related constraints on overwintering young-of-the-year striped bass (morone saxatilis). Can J Zool 79: 129–136.

5. Young P, Cech J (1993) Effects of exercise conditioning on stress responses and recovery in cultured and wild young-of-the-year striped bass, *morone saxatilis*. Can J Fish Aquat Sci 50: 2094-2099.

6. Videler J (1993) Fish swimming. Chapman & Hall, London, UK.

7. Beamish F (1978) Swimming capacity. In: Hoar W, Randall D, editors, Fish Physiology. New York: Academic Press, pp. 101-187.

8. Hammer C (1995) Fatigue and exercise tests with fish. Comp Biochem Physiol 112A: 1–20.

9. Plaut I (2001) Critical swimming speed: its ecological relevance. Comp Biochem Physiol A 131: 41–50.

10. Press W, Teukolsky S, Vetterling W, Flannery B (1992) Numerical recipes: the art of scientific computing: 3rd *Edition*. Cambridge Univ. Press, New York, NY, 345-346 pp.

11. Couzin I, Krause J, James R, Ruxton G, Franks N (2002) Collective memory and spatial sorting in animal groups. J Theor Biol 218: 1-11.

12. Dickey D, Wayne A (1981) Likelihood ratio statistics for autoregressive time series with a unit root. Econometrica 49: 1057-1072.

13. Zar J (1999) Biostatistical analysis, 4th. Upper Saddle River, New Jersey: Prentice Hall.
